# Lab Scale Closed-Loop Recycling of Polycarbonate Bioreactors for Sustainable Process Development

**DOI:** 10.1007/s00253-026-13796-z

**Published:** 2026-03-27

**Authors:** Magali Barbaroux, Eric Sorge, Pierre Moulinié, Jannik Dippel, Janice Zitoun, Roberta Chiara Tosato, Alessandro Vanni

**Affiliations:** 1 Sartorius Stedim FMT S.A.S., (Corporate Research) ZI Des Paluds, Avenue de Jouques, 13400 Aubagne, France; 2 Merck & Co., Inc., 126 E Lincoln Avenue, Rahway, NJ 07065 USA; 3 Covestro LLC, 1 Covestro Circle, Pittsburgh, PA 15205 USA; 4 Sartorius Stedim Biotech GmbH, August-Spindler-Strasse 11, 37079 Göttingen, Germany; 5 Sartorius Stedim Italy S.R.L. , Via A. Meucci N.4, 50012 Grassina, Bagno a Ripoli, Italy; 6 The Automation Partnership (Cambridge) Limited, Part of the Sartorius Stedim Biotech Group York Way, Royston, Hertfordshire SG8 5WY UK

**Keywords:** Cell-culture, Bioreactor, Plastic, Recycling, Polycarbonate, Life-cycle-assessment, Circular-economy

## Abstract

**Abstract:**

Despite well-known benefits, the increasing use of single-use technology (SUT) in biopharmaceutical processes has raised concerns about the environmental impact of plastic waste. This paper provides the first bioprocessing industry example of a “closing the loop” proof of concept by implementing a circular economy model for a polycarbonate (PC) bioreactor vessel used in process development applications. Through a collaborative effort between an end user, a SUT supplier, and a resin supplier, a lab-scale study was initiated to collect, decontaminate, and mechanically recycle material from vessels after use in mammalian cell culture experiments to produce new vessels. The study demonstrates that using recycled PC reduces vessels' environmental footprint and does not adversely impact vessel extractables. Even in direct contact with cells and media, recycled PC left cell culture performance and monoclonal antibody production largely unaffected. This work paves the way for broader adoption of circular practices in the industry. Life Cycle Assessment (LCA) was used to evaluate closed-loop recycling of PC across multiple environmental indicators (e.g., climate change, resource use, water use, etc.). Benefits typically favor the closed-loop system, but sensitivity analysis indicates that variations in parameters such as recovery yield, contamination rate, means of transport, and electricity mix can erode these advantages in non-ideal settings. The paper outlines the logistics, challenges, and learnings from this program, emphasizing the need for standardized procedures and collaboration across teams to achieve sustainable SUT circularity.

**Key points:**

• *Recycling bioreactor polycarbonate vessels reduces environmental footprint.*

• *Equivalent extractables observed in virgin and recycled polycarbonate vessels.*

• *Cell culture performance is comparable across recycled and virgin vessels.*

**Graphical Abstract:**

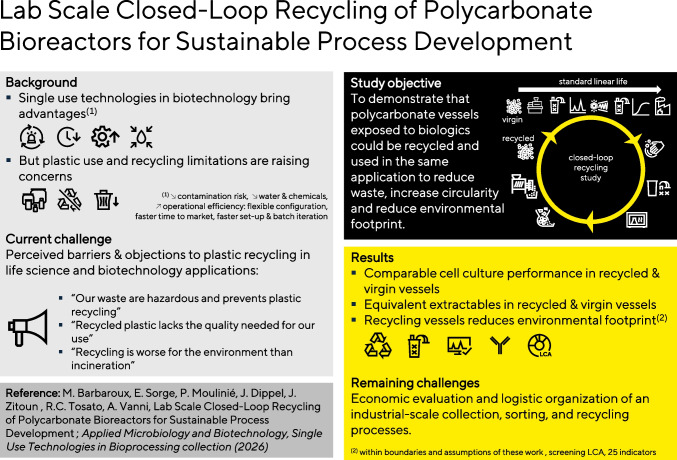

**Supplementary Information:**

The online version contains supplementary material available at 10.1007/s00253-026-13796-z.

## Introduction

Over the past twenty years, innovation in single-use technologies in the biomanufacturing industry has significantly boosted upstream bioprocessing capabilities for several applications, from mammalian cell cultures to microbial fermentation, and more recently advanced therapies (Rotondi et al. 2021) and industrial biotechnology. This transition to single-use components made from plastics has increasingly replaced traditional glass and stainless-steel bioreactors across a wide range of volumes, from small-scale benchtop bioreactors to large manufacturing instruments. Single-use bioreactors reduce complexity, setup times, and costs, resulting in greater process adaptability and faster time to market, along with environmental advantages such as lower water and energy usage (Pietrzykowski et al. 2013; Galliher 2018; Eibl and Eibl 2019; Budzinski et al. 2022).

Small-scale, automated, multiparallel bioreactor systems have become essential tools in early-stage bioprocess development. Every new batch or experiment begins with a fresh set of vessels which are provided irradiated and ready for inoculation, minimizing risks of cross-contamination from previous runs, require minimal assembly/disassembly, facilitating aseptic operations during their use, and removes the need for complex cleaning and sterilization procedures in the lab. Benefits of such a setup include quicker transitions between experiments, better productivity, and decreased downtime. Additionally, the elimination of cleaning and sterilization leads to lower water consumption and reduced use of cleaning agents. Lab-scale bioreactor vessels are typically manufactured from polycarbonate (PC) to meet essential requirements such as optical clarity, resistance to heat and impact, and long-term stability—as well as compatibility with various sterilization techniques. Once used, PC vessels are most often treated as hazardous waste, like other consumables exposed to biological materials. They are discarded via landfill or incineration, with or without decontamination, depending on prevailing regulations or organizational protocols.

Increasing environmental concerns on climate change and plastic pollution have led both governmental and non-governmental organizations to advocate for greater circularity in the plastics industry, with ongoing discussions around recycling definitions and hierarchies. Mechanical recycling of plastics into new articles or as a material stream into formulated materials is generally favored as it has been practiced in numerous unregulated industries for many years. The life sciences sector is also pursuing more circular approaches to address the challenge of waste from SUT (Ottinger et al. 2022; Luu et al. 2022; Barbaroux et al. 2024), though progress is limited by two key challenges: (1) local waste-management regulations on products that have come into contact with biological materials limit recycling opportunities (HPRC 2023) and (2) concerns about the consistency and safety of recycled plastics, particularly regarding potential extractables when used in biologics-related applications (Alassali et al. 2021; Schyns and Shaver 2021; Cecon et al. 2021; Lee et al. 2025).

**Fig. 1:**
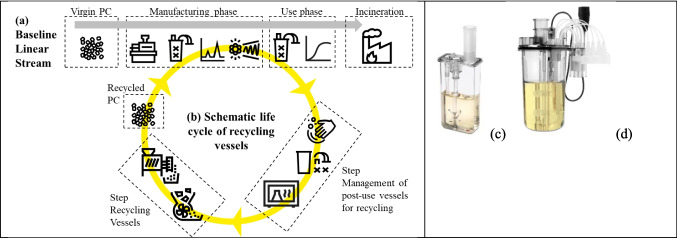
Schematic life cycle of recycling vessels (a) Baseline Linear Stream: “Virgin PC” means “not used or not processed before PC”; the “Manufacturing phase” includes injection molding, assembling, irradiation, and sensor calibration; the “use phase” includes cell culture and protein production; the vessels are then discarded by incineration; ideally with energy recovery; landfilling was deliberately excluded as a disposal option; despite its relatively low carbon footprint, it remains the least preferred waste-management method in Europe. b) Schematic life cycle of recycling vessels: the step “management of post-use vessels for recycling” includes cleaning, disconnecting, autoclaving; the step “recycling of vessels” includes – grinding, extruding, pelletizing; the recycled PC then undergoes the same manufacturing and use phases as the reference. (c) Bioreactor Ambr® 15 Cell Culture vessel with a maximum working volume of 15 mL. (d) Bioreactor Ambr® 250 High Throughput (HT) mammalian vessel with a maximum working volume of 250 mL.

This study aims to delve into the latter and investigate the technical feasibility of mechanical recycling 1of PC vessels that have been exposed to biologics and reusing them in the same application following a closed-loop recycling model established between a SUT bioreactor supplier and an end user (Fig. 1). The focus is on products used in research and development settings, where material changes are subject to fewer constraints compared to a strictly regulated manufacturing environment.

While most plastic recycling research has focused on how processing affects physical and mechanical properties (Pérez et al. 2010; Barbaroux et al. 2024; Khang Nguyen et al. 2025; Petousis et al. 2025; Moulinié et al. 2022), this study aims to explore the chemical and biological factors that may be influenced by the quality of recycled vessels in a closed-loop system. Specifically, it focuses on the material and functional quality of vessels composed of post-use recycled PC, with attention to extractables and leachables as well as the vessels’ ability to support the same cell culture and protein production as those made of virgin material.

A Screening Life Cycle Assessment (LCA) was conducted to evaluate the environmental impacts of the product’s life cycle under study, and to investigate if recycling could be a potentially viable option for the product. This included the lab-scale study of decontaminating and collection from the end user to the processing site.

## Material and methods

### Materials and processes

This study focuses on making 15 mL sparged microbioreactors, known as Ambr® 15 Cell Culture vessels (Item no.: 001-7B01 in Fig. 1(c)) with recycled material. These vessels have a higher surface-to-volume ratio of 2 cm^2^/mL—compared to 0.95 cm^2^/mL for larger formats—which makes them a worst-case for testing extractables and cell compatibility. To gather enough material, larger 250 mL vessels, known as Ambr® 250 HT (High Throughput) mammalian vessels (Item no.: 001-5G25, Fig. 1(d)), were recycled after use in mammalian cell culture experiments. The vessels were made from transparent medical grade PC (Makrolon® Rx2430 from Covestro) with a molecular weight (MW) of approximately 24.0 × 10^3^ g/mol, determined by GPC in THF with PS standards. The vessel includes polypropylene (PP) components, like the impellor and sparger, and adhesive-mounted optical pH and DO sensor patches for non-invasive measurement 2.

Ambr® 250 HT vessels are exposed to blends of nutrient-rich media and feeds, in addition to therapeutic molecules of various Occupational Exposure Bands (OEB), during their use in bioprocess development experiments. In the present study, only those Ambr® 250 HT vessels that were exposed to molecules below OEB4 and OEB5, the designations for highly hazardous and potent compounds^3^, were collected for recycling. Approximately 300 Ambr® 250 HT vessels were collected to exceed the minimum mass of 20 kg required for lab-scale recycling process.

To enable recycling, a standard fed-batch reactor harvest and takedown workflow was modified with additional steps for sterilization. As is typical, unharvested cell culture fluid was deactivated by adding bleach to 10% of the vessels’ liquid volume and holding for 10 min. Fluid was then safely poured down an approved drain. A small volume of tap water was added to fully harvested reactors, which contained only culture residue, before bleach deactivation. Foam and cell debris, whether bleached or unbleached, often remained in the reactors. The multi-component, multi-material reactor lids, including the agitator, pH probe, and gas lines, were discarded as regulated medical waste, leaving only PC vessels. Tap water was added to the vessels and poured down the drain to remove the bulk of foam and bleached cell debris. Residual cell debris was wiped with disposable lab towels and discarded in a biohazard bin. Vessels were rinsed a final time with half a vessel volume of tap water. Any remaining biohazard risk was eliminated by autoclaving vessels on-site at 121 °C and high pressure for 30 min. Once autoclaved, the vessels met Environment, Health, Safety (EHS) and shipping requirements for standard shipping to the off-site recycling facility as non-biohazard materials.

DO and pH patch sensors were removed from the vessels prior to grinding with pre-cleaned equipment. Ground flakes were subsequently pelletized by extruding the flakes through a twin-screw extruder equipped with a strand pelletizer and collecting the pellets. Shredding of the sterilized (used) vessels was performed with a pre-cleaned CONAIR WOR-TEX shredder. Pelletization was done by extruding the PC on a Coperion 25 mm twin-screw extruder with rates of either 15 or 24 kg/h. The MW of the pelletized material was approximately 22.4 × 10^3^ g/mol.

Recycled pellets were received by Sartorius Stedim Lab Ltd. (UK) where, along with virgin pellets, they were converted on the regular manufacturing equipment into Ambr® 15 mL bottom vessels with recycled contents of 0% (virgin as a reference), 20%, 50%, and 100% to define a maximum recycled content threshold for the application, if any. Standard injection molding parameters were used. Then, vessels were incorporated into ready-to-use 25 kGy e-beam sterilized Ambr® 15 vessels with standard manufacturing conditions, with impellers, sampling tips, and patch sensors. Impellers and lids were manufactured with virgin PC.

### Extractables

Extraction involves obtaining an extract from a liquid or solid using a suitable solution. Efficiency is influenced by factors like diffusivity in the polymer, temperature, contact time, mixing, surface-to-volume ratio, and extraction agent choice. The process used pure ethanol under worst-case conditions to maximize potential extractables detection, offering a conservative estimate of extractable profiles (Gilleskie et al. 2021). Pure ethanol was selected to ensure the highest sensitivity and selectivity for disposable systems regarding extractable substances (Dorey et al. 2018). Our results can be seen as a worst-case approach for various cultivation and fermentation processes. Extractions followed 10,993–1 standards (Thangaraju and Varthya 2022). Extractable trials were carried out using vessels with 100% recycled PC, acknowledging that a 100% recycling rate is not feasible due to unavoidable material losses over the product life cycle and less-than-complete return rates, as will be elaborated in the screening LCA sections. Triplicates of virgin and recycled vessels with a 63 cm^2^ inner contact surface were filled with 15 mL ethanol, achieving a 4:1 cm^2^/mL surface-to-volume ratio, sealed with PTFE tape, and extracted at 40 °C and 75 rpm for 21 days. The PC lid was not in direct contact with the extraction medium. Around 3 cm^2^ of the PC impellor was in contact with the extraction medium. Therefore, the recycled content in the vessels used for extraction trials is more than 95%. Various chromatographic tests were used for analytical determination due to numerous plastic additives and extractable compounds. Data is documented in µg/cm^2^ or at the reporting limit if below detection (Scott 2007). The reporting limit, related to method sensitivity, is the lowest concentration measurable with precision (Scott 2007). According to USP 665, substances are reported that are above the reporting limit. For the HPLC–UV the reporting limit is 0.08 µg/cm^2^, for the GC–MS and LC–MS the reporting limit is 0.03 µg/cm^2^ (Scott 2007). The methods used are based on the publication of Menzel et al*.* (Menzel et al. 2024), equipment details are provided in Supplemental, Material and methods.

### Cell Culture and Monoclonal Antibody (mAb and bsAb) production

Cell culture studies were carried out using recycled vessels to compare process performance and product quality between virgin and post-use, recycled reactors. The same proprietary fed-batch bioprocess was executed across 24 vessels composed of either 0% (virgin), 20%, 50%, or 100% recycled PC content. Two proprietary Chinese hamster ovary (CHO) cell lines producing, respectively, a monoclonal antibody (mAb) and a bispecific monoclonal antibody (bsAb) were used 4. For each cell line, a single seed train provided a common inoculum source for each recycled content condition. All 8 conditions (cell line x recycled content of vessel) were run in technical triplicates (n = 3 parallel bioreactors per condition) to assess vessel-to-vessel variability within each grade of recycled content (0%, 20%, 50%, and 100%). The 24 vessels were randomly distributed across the cell lines’ respective culture stations. Material-driven process performance was thus tested under controlled biological input 5. Standard process parameters, culture health metrics, and metabolite levels were monitored throughout the run using online vessel sensors and offline sampling. 1 mL culture samples were also collected on days 7, 9, 11, and 14 of the process for Protein A (ProA) titer analysis. Cell culture fluid was harvested on day 14 of the process and purified via ProA purification. A standard panel of product quality analyses was performed on ProA purified material (PAP) from each reactor to determine product purity in size, mass, and charge, as well as glycosylation patterns. UP-SEC, N-glycan, IEX, icIEF, and CE-SDS methods optimized for the mAb and the bsAb were carried out. Equipment details are provided in Supplemental, Material and methods.

### Trace element analysis

Trace metals serve important functions in maintaining cellular health and mature protein production. They are added to reactor vessels during cell culture processes via chemically defined culture media. These trace metals include common enzymatic cofactors and redox-active species such as manganese (Mn), iron (Fe), copper (Cu), zinc (Zn), and selenium (Se) (Ritacco et al. 2018). Batch-to-batch consistency of trace metal levels is important in bioprocesses because cell culture performance and protein production are sensitive to deviations in cofactor availability and redox balance (Graham et al. 2019). A trace element study was therefore conducted to determine whether these trace metals, which were present in the source PC’s original cell cultures, leach from recycled PC vessels under culture-like conditions. To minimize background trace element levels, ultrapure water was incubated in unused virgin and recycled vessels under conditions that mimicked those experienced during cell culture. 14 mL of sterile, ultrapure water was incubated in duplicates of vessels of each recycling grade (0%, 20%, 50%, and 100% recycled PC). The 8 vessels were loaded onto a microscale bioreactor workstation and incubated for 14 days at a high 38 °C. Online pH was maintained between 5.95 and 7.20 using mild CO2 and O2 sparging. Agitation was set to 900 rpm for the duration of the incubation. Following the incubation period, the water samples were prepared for analysis via acidification (without microwave digestion) with a trace amount of nitric acid. Analysis was carried out via inductively coupled plasma mass spectrometry (ICP-MS). Data was further analyzed using a linear fit test to determine if trace element levels changed linearly with the proportion of recycled PC in the vessels. One-way ANOVA was also carried out to determine if there was a significant difference in mean trace element levels in any of the vessel types. Equipment details are provided in the supplementary file.

### Life Cycle Assessment (LCA)

The LCA methodology was selected to provide environmental impact data for the life cycle of the 250 mL vessel. The LCA object is the Ambr® 250 High Throughput (HT) mammalian vessel, comparing two scenarios: one using virgin PC only and another using post-consumer recycled PC from closed-loop recycling for the main PC part of the vessel.

In this screening LCA, the PC vessel, excluding the lid and impeller, is sent for recycling. Based on the data and assumptions, including process yield, the available PC at End of Life can theoretically meet the material needs for a vessel with 90% recycled content maximum. Different conditions could lead to varying outcomes.

The declared unit of this LCA is a 250 mL vessel with packaging, covering cradle-to-grave system boundaries, including material extraction, production, sterilization, distribution, and end-of-life treatment, with recycling processes for closed-loop PC vessel recycling. Primary data on the bill of materials and vessel component manufacturing were used, considering scrap rates for injection molding. The manufacturing site is in England. Environmental burdens of virgin PC were sourced from the Sphera® MLC database. When primary data were unavailable, literature and databases like Sphera® MLC v2025.2 and ecoinvent v3.10 were used. Manufacturing scraps are excluded from closed-loop recycling. Sterilization and autoclaving data came from IBA Industries and McGain et al. 2017. Assembly/disassembly of vessels was considered manual. Distribution is assumed via sea shipments and trucks to Merck & Co., Inc., 126 E Lincoln Avenue, Rahway, NJ, 07065. PC parts take-back is via trucks. Recycling of used vessels occurred in the US, using primary data. Regrinded granules with recycled content were shipped via sea and road from the recycler to the manufacturing plant. Non-recycled vessel components were incinerated. The study analyzes scenarios comparing vessels made of virgin PC, and those with 90% and 20% recycled content. Calculations were made using Sphera LCA for Expert software, v10.9.3, with the EF v3.1 LCIA method.

The scenarios for the 250 mL vessel include a baseline using virgin vessel, steam sterilized, and incinerated, and two closed-loop options: vessels with 20% and 90% recycled content (Table 1). Other recycled content rates fall between these options. Additional information is provided in the supplemental file, including the Life Cycle Inventory for the main processes (Table S1).

**Table 1:** Screening LCA scenarios of virgin and recycled PC vessels closed-loop recycling

Scenarios	Reference	Closed-loop – 20%	Closed-loop – 90%
Content of recycled	0%	20%	90%
Content of virgin	100%	80%	10%
Water to recycle	NA	0.08 L	0.08 L
Energy to recycle	NA	0.34 kWh/kg	0.34 kWh/kg
Losses in recycling	NA	1.5%	1.5%

## Results

### Materials and recycling

**Fig. 2:**
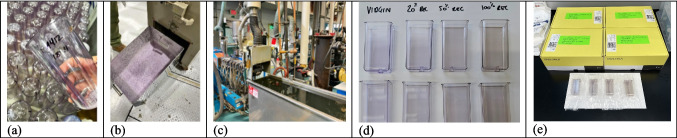
a a) Used/sterilized PC vessels prior to grinding; (b) PC flakes from ground vessels; (c) extrusion line used for pelletization of PC flakes; (d) recycled vessels from left to right with 0%, 20%, 50%, and 100% recycled PC content; (e) vessels after 25 kGy e-beam irradiation with 0%, 20%, 50%, and 100% recycled PC content

Figure 2 (a) to (e) illustrates the conversion of collected 250 mL vessels into pelletized material that was used to injection-mold “new” 15 mL vessels for this study. The process had little loss of material (< 2%) and is amenable to be done at higher scale (up to 100 kg/h) if necessary. Lower rates of no more than 25 kg/h were used on account of the quantity of material to be recycled. The melt flow and molecular weight measurements illustrate our minor degradation introduced by the process: compared with the virgin material, the recycled PC processed at 24 kg/h exhibits an increased Melt Volume Rate (from 19 to 26 cm^3^/10 min at 300 °C/1.2 kg) and a slight decrease in molecular weight (from 24 340 to 22 650 g/mol).

The recycled materials were dry blended with virgin resin of the same grade to achieve 0%, 20%, 50%, and 100% recycled PC content. Afterwards, 15 mL vessels were manufactured via injection molding using manufacturing equipment and process parameters. Assembly, calibration, packaging, and e-beam irradiation with a dose of 25 kGy were done according to standard manufacturing parameters. The recycling content did not have any impact on manufacturability. All samples were produced without any scrap waste. As described in Fig. 2, the only discernable difference from increasing the content of recycled PC was the color shift.

### Extractables

Table 2 lists detected substances in µg/cm^2^. " < RL*" denotes concentrations below the reporting limit, indicating levels too low for reliable quantification. Total extractables per vessel are calculated by multiplying surface-specific values by the 63 cm^2^ effective surface area, matching the 15 mL standard working volume. Substances were detected in similar amounts in recycled and virgin vessels, with no agglomeration of extractables from recycling.

**Table 2:** Concentrations of detected extractables compounds in vessels containing 100% recycled content and toxicological thresholds for safety assessment

Compound	CAS Number	PDE/TTC [µg/day]	Quantity/EFA/Surface [µg/cm^2^]		Detection method
			Virgin vessel	Recycled vessel	
4-Hydroxyacetophenone	118–93-4	120	< RL*	< RL*	GC–MS
p-tert-Butylphenol	98–54-4	120	< RL*	< RL*	GC–MS
Phenol	108–95-2	12,500	< RL*	-	GC–MS
Chlorobenzene	108–90-7	3600	1,30	1,03	HPLC–UV
Palmitic acid (C16:0)	57–10-3		0,22	0,30	LC-HRMS
Stearic acid (C18:0)	57–11-4	120	0,23	0,29	LC-HRMS
Branched Alkane	-	120	< RL*	< RL*	GC–MS
Diacetone alcohol	123–42-2	120	0,17	0,15	GC–MS
1,3-Di-*tert*-butylbenzene	1014–60-4		0,03	0,03	LC-HRMS
Diethylene glycol	111–46-6	120	< RL*	< RL*	GC–MS
Diethylene glycol, bis TMS derivate	16,654–74-3	120	0,3	0,3	GC–MS
Adhesives from the Patch	13,622–90-7; 17,901–48-3; 18,937–00–3	120	0,59	0,54	GC–MS
2,4-Di-*tert*-butylphenol	96–76-4	120	0,04	0,05	LC-HRMS
Cyclohexanol	108–93-0	120	0,06	0,05	GC–MS
2-Ethylhexanol	104–76-7	120	< RL*	< RL*	GC–MS
2-Methylheptanoic acid	1188–02–9	120	< RL*	< RL*	GC–MS

^*^ Reporting limit.

### Cell Culture and Monoclonal Antibody (mAb and bsAb) production

Across vessel recycling grades (0%, 20%, 50%, 100%), cell culture performance and product quality results were comparable for most measured attributes in both the bsAb and mAb. Vessels composed of 0% recycled PC served as controls and exhibited acceptable process performance and product quality with respect to both cell lines’ historical data and clinical and/or commercial release specs. Across recycled contents, the magnitudes of product quality variations were minimal, and those attributes for which statistically significant linear trends were observed are discussed further. Interpretations of the data presented in this section can be found in the Discussion section.

**Fig. 3:**
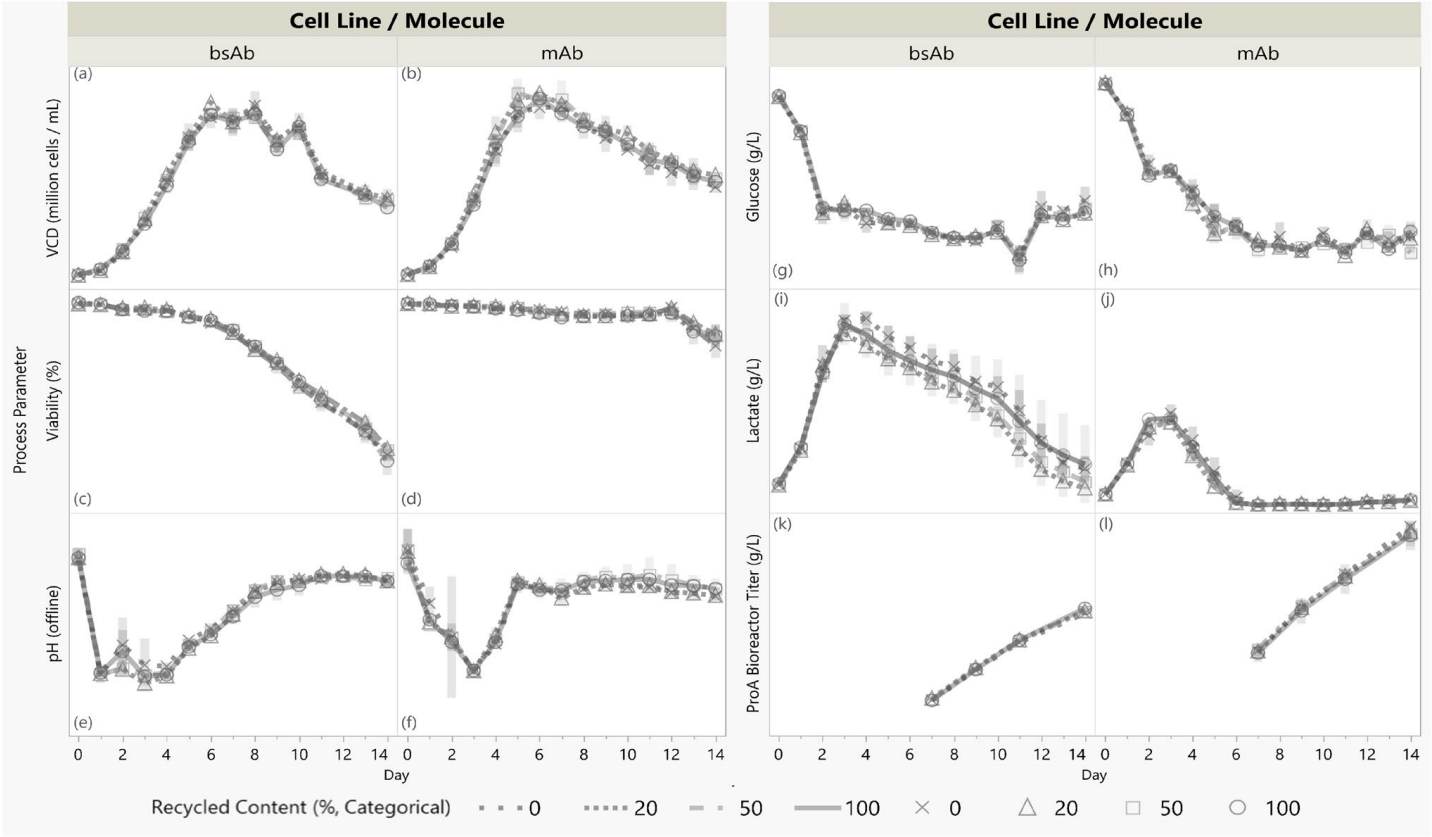
Process data over time (days) from mAb and bsAb cultured in 15 mL vessels composed of various proportions of recycled material (%). Points (shapes indicated in the legend) represent mean values; shaded regions show ± 1 standard deviation from the mean. N = 3 for all conditions except bsAb 20% recycled (N = 2) due to omission of an outlier reactor wherein the culture experienced runaway lactate, which may result from improper CO_2_ stripping due to possible airline obstruction. Y axis values are omitted to protect proprietary data; raw data trends are shown for visual comparison

For both cell lines and across recycling grades, mean values for viable cell density (VCD), viability, pH, glucose, and lactate followed comparable trends over process time. Most daily means were within one standard deviation across recycling grades (Fig. 3 a-j). Titer trends were also comparable between reactor types (Fig. 3 k-l). Product quality attributes were analyzed using linear regression with recycled content treated as continuous (0–100%). Reported metrics include R^2^, to reflect the extent of each attribute’s fit to the lines of fit against recycled content, and p-values from F tests for trend significance. In all linear fit analyses, the tested null hypothesis was that the true slope of the product quality attribute over % recycled polycarbonate was zero. A significance level of 0.05 was used, meaning that a p-value below 0.05 led to a rejection of the null hypothesis and thus indicated a statistically significant linear trend between the quality attribute and recycled content. One-way ANOVA analyses, with categorical recycled contents (0%, 20%, 50%, 100%), were performed as well; the one-way ANOVA and p values resulting from both the linear fit tests are visualized in the supplementary file, Fig. S1 and Fig. S2 respectively.

**Fig. 4:**
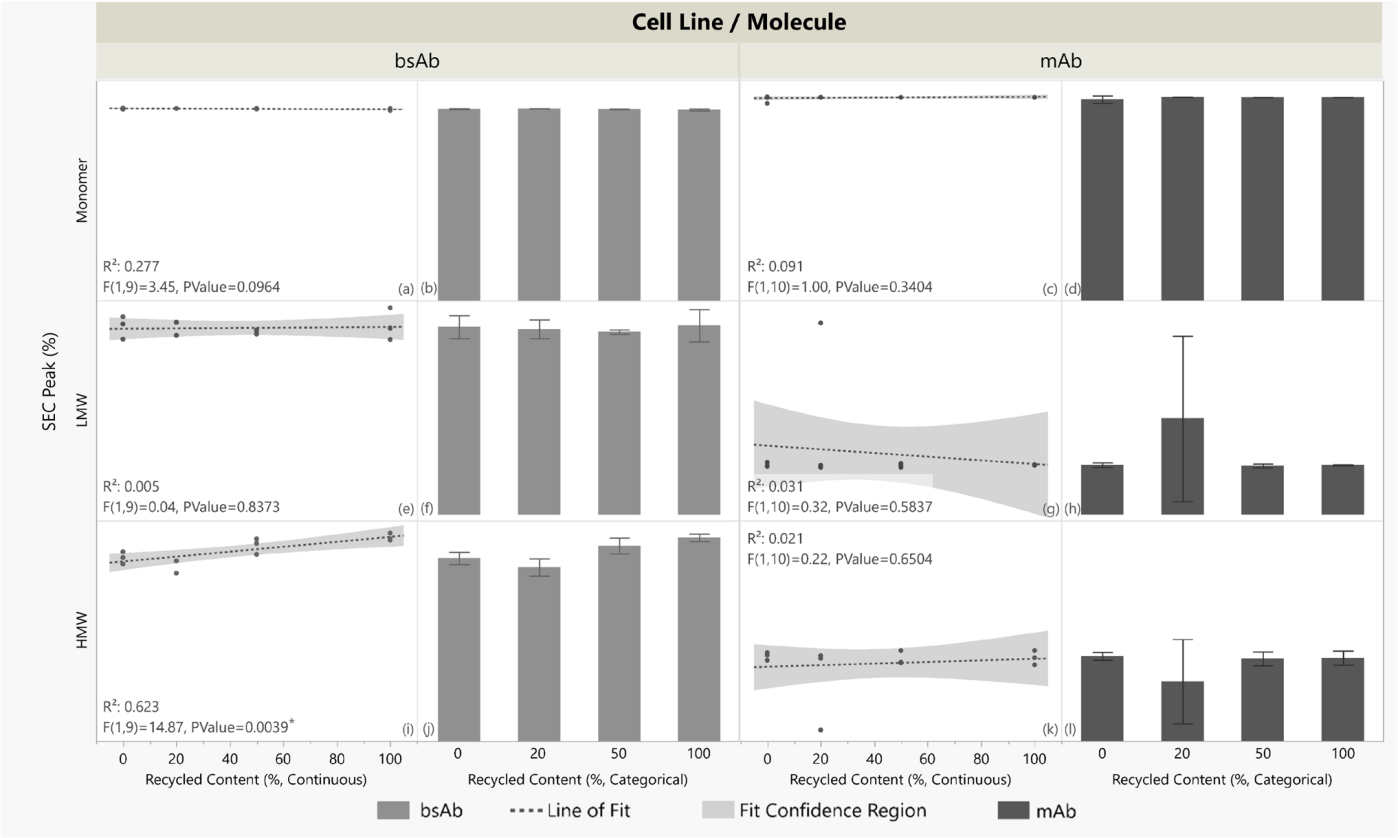
Size Exclusion Chromatography (SEC) peaks (%) versus recycled content (%) for bsAb and mAb. Panels a, c, e, g, i, and k: recycled content treated as continuous (0–100%); linear fit (dashed) models and 95% confidence bands (shaded) are shown (R^2^, P values annotated). Asterisks (*) indicate the significance of a trend. Panels b, d, f, h, j, and l: mean peak values are shown with ± 1 standard deviation error bars; recycled content is categorical with four distinct classes. N = 3 except for bsAb 20% (N = 2) due to outlier reactor omission. Y axis values are omitted to protect proprietary data; raw data trends are shown for visual comparison

Figure 4 shows monomer and low molecular weight (LMW) peaks did not change significantly with recycling grades for both the mAb and bsAb. No significant trend was detected between mAb high molecular weight (HMW) peaks and recycled content; as is addressed in the Discussion section, however, bsAb HMW peaks increased with recycled content (p = 0.0039, R^2^ = 0.623).

**Fig. 5:**
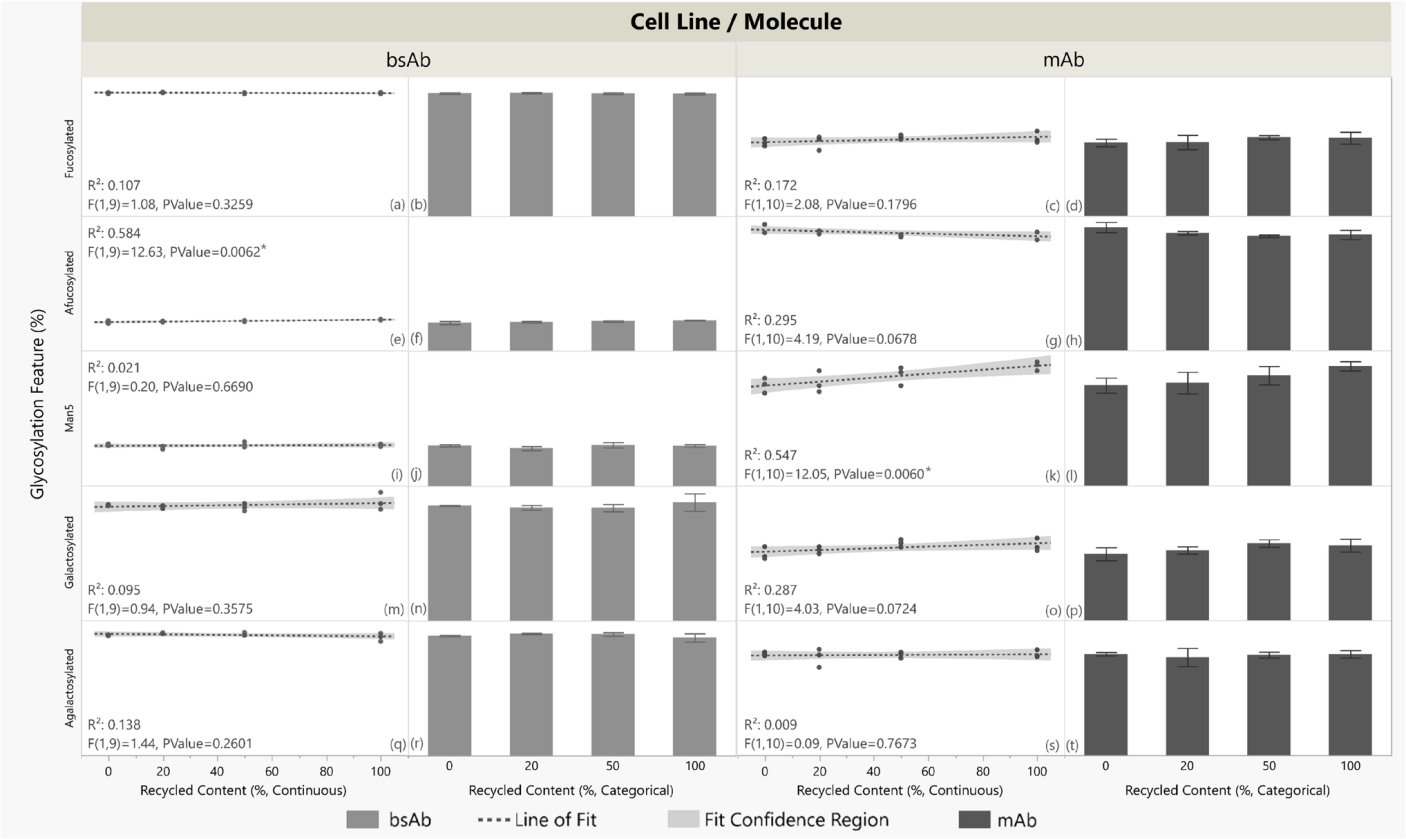
Glycosylation features (%) versus recycled content (%) for bsAb and mAb. Panels a, c, e, g, i, k, m, o, q, and s: recycled content treated as continuous (0–100%); linear fit (dashed) models and 95% confidence bands (shaded) are shown (R^2^, P values annotated). Asterisks (*) indicate the significance of a trend. Panels b, d, f, h, j, l, n, p, r, and t: mean peak values are shown with ± 1 standard deviation error bars; recycled content is categorical with four distinct classes. N = 3 except for bsAb 20% (N = 2) due to outlier reactor omission. Y axis values are omitted to protect proprietary data; raw data trends are shown for visual comparison

Figure 5 shows glycosylation profiles. For the bsAb, fucosylation, man5, galactosylation, and agalactosylation showed no statistically significant linear relationship with reactor recycled contents. The bsAb’s afucosylation levels exhibited a positive trend with recycled content (p = 0.0062, R^2^ = 0.584). The mAb fucosylation, afucosylation, galactosylation, and agalactosylation levels showed no statistically significant trend with recycling content with 95% confidence. The mAb man5 levels increased, however, with increasing recycling content (p = 0.0060, R^2^ = 0.547). One-way ANOVA (supplementary file Fig. S1) indicated no statistically significant differences in means among any glycan features across categorical recycling contents for both the mAb and bsAb.

**Fig. 6:**
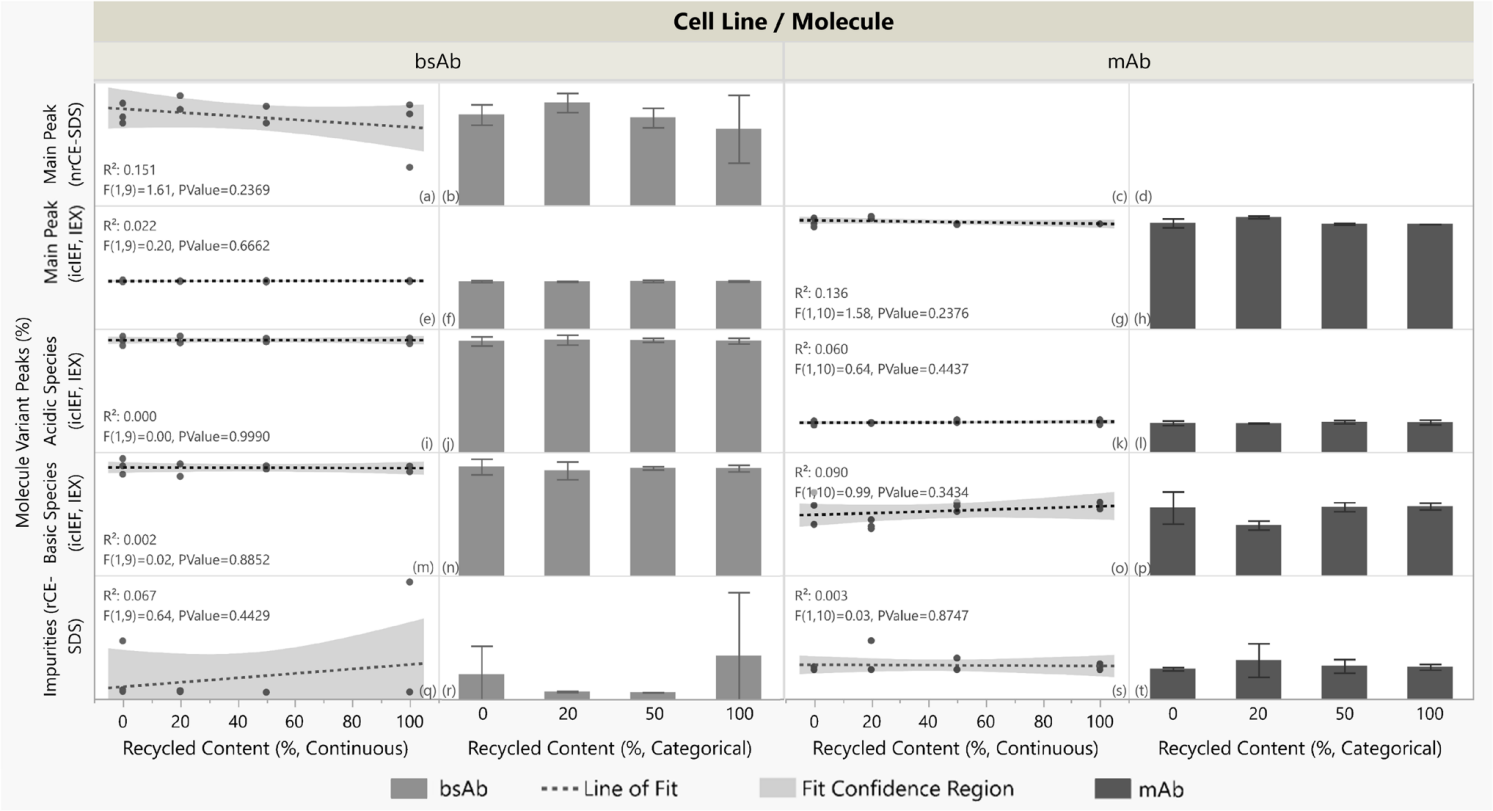
Molecule variant peaks (%) from CE-SDS (non-reduced [nr-] and reduced [r-]), icIEF, and IEX versus recycled content (%) for bsAb and mAb. Panels a, c, e, g, i, k, m, o, q, and s: recycled content treated as continuous (0–100%); linear fit (dashed) models and 95% confidence bands (shaded) are shown (R^2^, P values annotated). No significant trends were observed. Panels b, d, f, h, j, l, n, p, r, and t: mean peak values are shown with ± 1 standard deviation error bars; recycled content is categorical with four distinct classes. N = 3 except for bsAb 20% (N = 2) due to outlier reactor omission. No nrCE-SDS method for the mAb. Y axis values are omitted to protect proprietary data; raw data trends are shown for visual comparison

The mAb and bsAb characterization profiling from CE-SDS, icIEF, and IEX results showed no significant linear relationships between recycled content and product main peak, acidic species, basic species, nor impurities for either molecule (Fig. 6).

### Trace elements

**Fig. 7:**
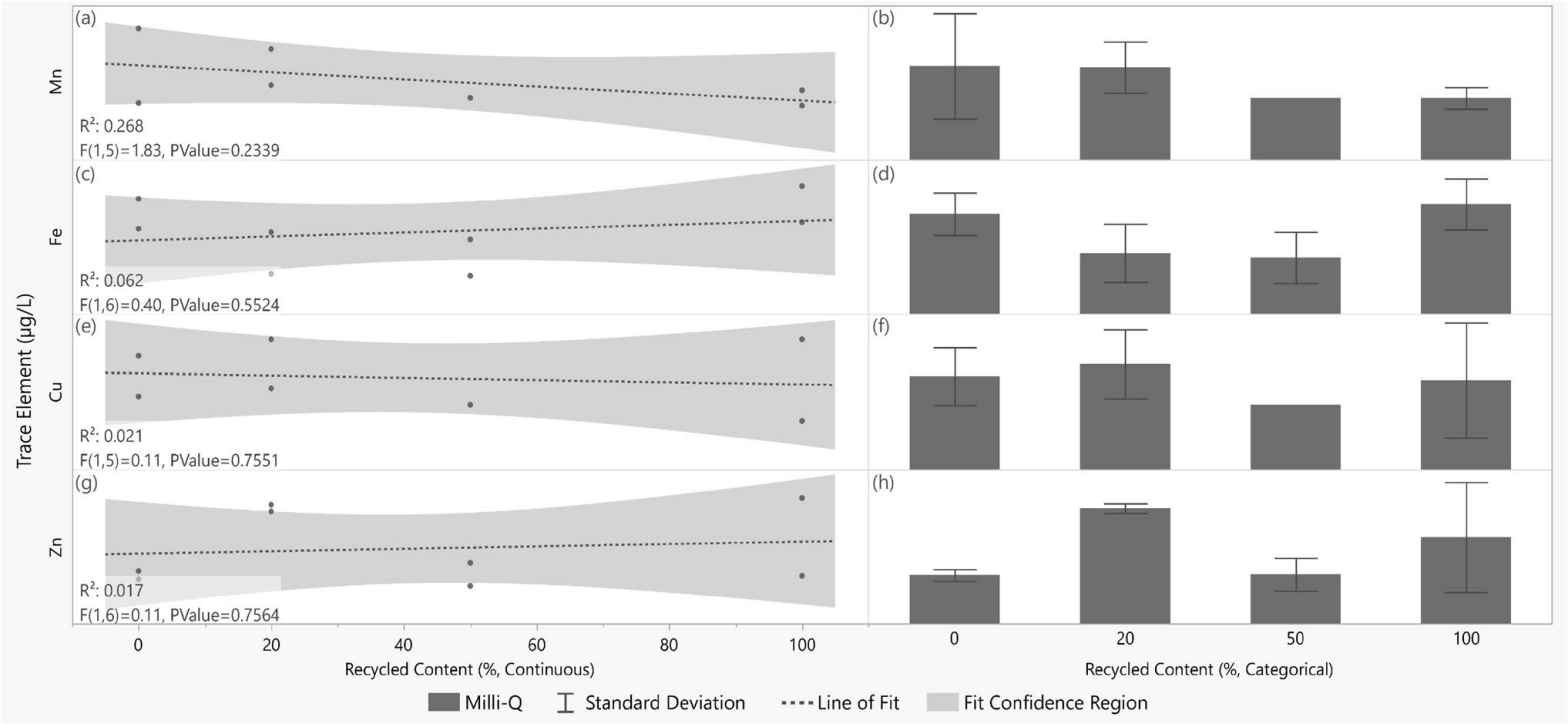
Trace element levels (µg/L) from ICP-MS versus recycled content (%) for incubated water samples. Panels a, c, e, and g: recycled content treated as continuous (0–100%); linear fit (dashed) models and 95% confidence bands (shaded) are shown (R^2^, P values annotated). Panels b, d, f, and h: mean peak values are shown with ± 1 standard deviation error bars; recycled content is categorical with four distinct classes. Se showed no signals above the limit of detection and is therefore not plotted. Y axis values are omitted to protect proprietary data; raw data trends are shown for visual comparison

Manganese (Mn), iron (Fe), copper (Cu), and zinc (Zn) were each detected at low, near-limit-of-detection levels across all samples. ICP-MS results (Fig. 7) showed no statistically significant difference in trace element mean levels between vessel types, nor any linear relationship with the proportion of recycled material composing the 15 mL vessel. Selenium (Se) levels were below the limit of detection for all samples.

### Life cycle assessment (LCA)

**Fig. 8:**
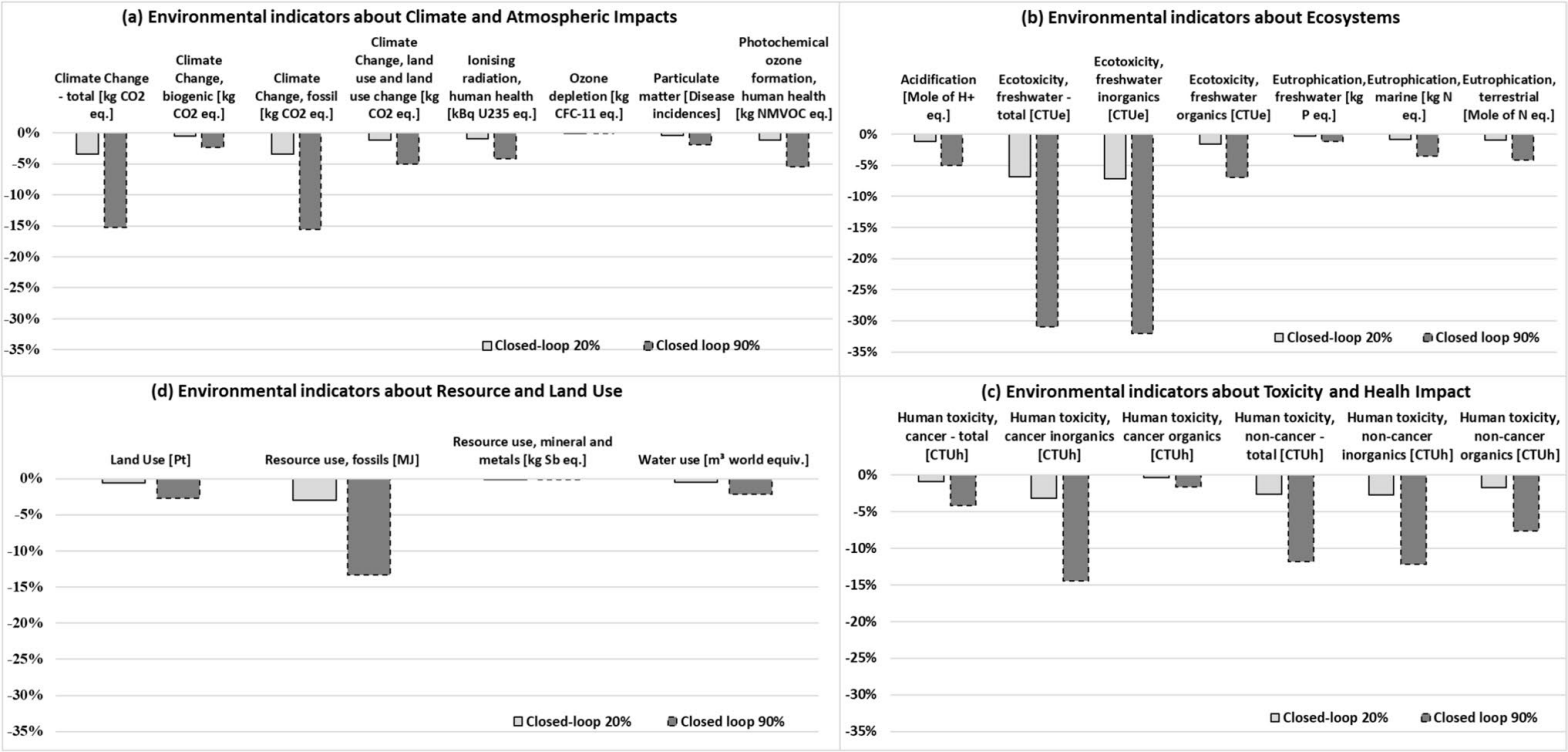
Difference between the baseline and the scenarios analyzed: Closed-loop – 20% and Closed-loop – 90%. Life Cycle Impact Assessment results obtained by applying EF v3.1, grouped as follows: (a) Climate and Atmospheric impacts, (b) Ecosystems, (c) Toxicity and Health impacts, (d) Resource and Land Use. Mode of transport: ship and truck

Figure 8 shows the difference from the baseline for improved scenarios (Closed-loop – 20% and Closed-loop – 90%) using the EF method, grouped by type of environmental indicators (Climate and Atmospheric impacts, Ecosystems, Toxicity and Health impacts, Resource and Land Use) for clarity. Calculations indicate that all improvement scenarios have less impact than the baseline for every indicator. Within this study, recycling and creating a closed loop, using recycled material as secondary raw material for vessels, reduce environmental impacts by eliminating incineration and using less virgin raw material. Comparing Baseline to Closed-loop scenarios and being conscious of the potential uncertainty of the results, the impact categories assessed do not show a systematic worsening of the environmental impacts. Further information is available in the supplementary file.

## Discussion

### Materials and recycling

To enable PC vessel recycling, simple steps were added to the reactor takedown workflow on harvest day for sterilization. Novel workflows for waste diversion in circularity efforts must be simple, safe, and repeatable to ensure viable adoption.

Preparing vessels for diversion from the biohazard bin involved simple but laborious steps in this lab-scale program. Pouring bleached reactor contents through each lid's small opening caused foaming, requiring extra water rinses. Removing lids requires holding reactors upside down and applying palm force below gassing ports, posing a splash and breakage risk. The workflow was suitable for collecting about 300 vessels for lab-scale recycling's 25 kg requirement. At scale, a streamlined process addressing the de-lidding bottleneck would be more appropriate.

Recycling efforts, like this one, show potential for waste reduction and diversion in biopharmaceutical R&D. Accessible, easy-to-adopt workflows are needed for widespread circularity, requiring iteration and collaboration between material users and suppliers. Early, labor-intensive workflows serve as proof-of-concept trials, but broader participation and focus on bottlenecks will help develop optimized waste-diversion strategies.

The color change in recycled vessels was previously documented and did not impact pH and DO sensor calibration and control (Barbaroux et al. 2024). The minor decrease in viscosity and molecular weight from recycling PC vessels aligns with other findings (Moulinié et al. 2022), reflecting the material's two heat histories: original molding and extrusion/pelletization. MW values indicate that high molecular weight persists after processing. These decreases in viscosity and molecular weight have no effect on the manufacturability of the vessels.

Extractable substances were found in both virgin and recycled PC vessels, with no increase in concentrations in recycled vessels compared to virgin ones within detection limits. This indicates that recycling, including irradiation, autoclaving, and secondary manufacturing, does not negatively impact the extractables profile under worst-case conditions. Extractables may originate from PC, PP, or sensor patches, complicating source assignment. Extractables related to PP components included branched alkanes, stearic acid, and palmitic acid, consistent with PP additives and degradation products. Although Irgafos® 168, a PP antioxidant, was not directly observed, 1,3-Di-tert-butylbenzene, a degradation product (Maier and Schiller 2016), was detected, likely from the impellor and sparger. Diethylene glycol and derivatives were linked to adhesive residues from sensor patches. Despite these substances, recycled vessels did not show higher concentrations than new ones, suggesting that, within a scenario entailing proper collection, sorting and processing, closed-loop recycling is feasible.

It should be noted that this study considers only one recycling loop with two irradiation steps. Additional loops would further change the material through repeated heating and irradiation. Because the tests used 100% recycled polymers—an unrealistic rate for closed‑loop systems—mixing recycled with virgin materials is more practical. Such mixing improves physical and chemical properties and reduces risks from multiple recycling rounds, as the recycled fraction becomes diluted each cycle. Previous studies have calculated the equilibrium composition of recycled material after multiple recycling steps, showing that with a 30% recycling quote, the proportion of material recycled three times is below 2% (Barbaroux et al. 2024).

### Cell Culture and Monoclonal Antibody (mAb and bsAb) production

We assessed whether PC from used reactors can be recycled and reused in bioprocess experiments, specifically comparing the functionality of vessels made from recycled PC to those made from virgin PC. Due to material constraints in this lab-scale trial, used vessels were recycled and remolded into smaller vessels. Across recycling grades (0%, 20%, 50%, and 100%), two cell lines producing a bsAb and a mAb showed comparable process performance. Daily trends in growth, viability, pH, glucose, lactate, and titer overlapped across recycled contents, indicating that recycled vessels support similar culture performance to virgin PC vessels.

Subsequent product quality (PQ) testing of the bsAb and mAb PAP showed limited statistically significant association between PQ attributes and reactor recycled content.

Size exclusion chromatography (SEC), N-glycan assays, and protein characterization tests for purity and heterogeneity in charge and mass (icIEF, IEX, and CE-SDS) were conducted on both molecules. Recycled content was analyzed as a continuous variable to assess changes in product quality with % recycled content. Most attributes showed no statistically significant linear trend with % recycled content; p values are summarized in Fig. S2 in the supplementary file. Differences in attribute means across recycled contents were also largely statistically insignificant; the visualization of p values from one-way ANOVA between recycling levels is also in the supplementary file, Fig. S1.

For the bsAb, most attributes showed no statistically significant trends: SEC monomer and LMW peaks, four of five glycosylation patterns, and all six peaks in icIEF charge variant analyses and reduced/non-reduced CE-SDS testing remained unchanged with recycled content. Two exceptions were noted: SEC HMW peaks and afucosylation showed positive correlations with recycled content in linear models, demonstrating possible bsAb sensitivity to aggregation and glycan processing. Other SEC purity indices were unaffected, suggesting HMW increases were minimal; SEC monomers and LMW peaks did not significantly change with % recycled content. One-way ANOVA analysis (supplementary file Fig. S1) found no mean differences in bsAb glycan features, including afucosylation, across reactor types, indicating modest sensitivities in aggregation and glycosylation.

For the mAb, no significant trend with recycled content was observed in the SEC, IEX, nor CE-SDS tests; product purity in size, mass, and charge remained unchanged with % recycled content. Five of six glycosylation features showed no trend, but Man5 levels increased with recycled content in the linear model. Man5, a sensitive marker, warrants monitoring in future tests. Despite the positive trend with % recycled content, Man5 did not exceed historical levels for the mAb’s fed-batch process in this study.

Overall, product quality, including protein purity and glycosylation, was largely comparable across recycled contents for both molecules. In the three instances of significant linear trends, R^2^ values indicate variance explained by the model, not causation. Reactor placement was randomized, and handling was careful; additional replicates in follow-up experiments would reduce variability and clarify associations between PQ attributes and recycled content. Observed glycosylation trends were modest when considering categorical analysis and historical data, and HMW increases did not affect other purity measures like SEC monomer and LMW peaks. The cell culture data from an idealized closed-loop scenario therefore supports the functional suitability of vessels made from recycled PC.

### Trace elements

Variability in trace metal levels in CHO cell culture has been noted in the literature to impact culture performance and protein production through various mechanisms. Heightened Mn, Zn, Cu, and Fe levels, whether from supplementation or leaching, have been shown to affect culture and product characteristics including productivity, lactate metabolism, apoptosis trends, and glycosylation (Graham et al. 2019). Here, results from fed-batch processes in recycled 15 mL vessels showed that culture performance was not significantly affected by vessel recycled content, with product quality showing similar comparability. Nonetheless, an additional experiment was conducted to detect possible leaching of trace elements from vessels under cell culture conditions as this may be relevant for more sensitive cell lines than those tested in the described cell culture experiment.

After 14 days of incubation at 38 °C with gassing and agitation, ICP-MS analysis of the incubated water samples showed no significant linear relationship nor difference in means between trace element (Mn, Fe, Cu, Zn, Se) levels and vessel recycled content. Thus, despite source material exposure to high trace element levels during past cell culture, vessels molded from recycled material showed no evidence of heightened trace metal leaching compared to those made of virgin PC.

Because the source polycarbonate has been exposed to diverse mixtures of media, feeds, cell lines, and biomolecules during past cell culture experiments, we expect that these trace element findings are broadly representative of polycarbonate sourced from typical biopharmaceutical process development environments. Water was selected as the incubated liquid to minimize background trace metal levels and thereby enable detection of minute trace element differences. The process parameters selected for this water incubation study (agitation, gassing, pH, process time) reflect standard fed-batch conditions, and the temperature was intentionally set to a high 38 °C, representing—along with the high material-to-solvent ratio of the small 15 mL reactor vessels—a “worst-case” extraction condition (Dorey et al. 2018). Within the scope of conventional fed-batch operation, we would not expect moderate adjustments to process parameters in recycled vessels to significantly impact dissolved trace metal levels.

Nevertheless, as relevant to distinct fed-batch processes, further water incubation studies spanning broader parameter ranges (e.g., higher temperatures, lower pH, longer duration, and controlled light exposure) would be valuable to confirm, refine, and expand upon these findings. In the case of diverse media compositions during cell culture (e.g., high levels of chelating species), ICP-MS detection of minor trace metal variations would be masked by intrinsic media levels, and effects of potentially deleterious leaching species would necessarily be indirectly assessed via culture performance and product quality analyses.

### Environmental impact

The LCA screening examined two scenarios: closed-loop recycling where PC vessels are recovered and recycled to provide 20% and 90% recycled granules for the vessel. In this study, calculations show that all improvement scenarios do not contribute to worsening the baseline situation. Recycling and establishing a closed loop, utilizing recycled material as secondary raw material for vessels, avoiding incineration and decreasing the use of virgin raw materials, do not lead to an increase of impacts.

Establishing closed-loop recycling relies on material availability. In this study, 90% recycled content was the maximum achievable based on process yields, but lower yields could decrease this percentage. Using recycled content may not always be feasible. Therefore, two additional scenarios were included in the analysis:

Open-loop: at the end of life, PC components are sent to recycling, exiting the study's system boundaries, with no recycled content used in manufacturing the PC vessel.

Open-loop (15% return rate) (15% is the upper value reference by Mallick et al. 2022 for Returpen™): at the end of life, 15% of PC components are sent to recycling, exiting the study's system boundaries, with no recycled content used in manufacturing the PC vessel.

In the two open-loop scenarios, benefits arise solely from avoiding PC incineration, and compared to the Baseline, considering that uncertainty could affect the variables considered during the End-of-Life, they do not differ much from the Baseline (supplementary file, Fig. S3). Here, the sole advantage arises from end-of-life savings, specifically in reducing incineration emissions. The plastic is repurposed into a different product, and the life cycle assessment aims to measure the environmental impacts of a single product, making it challenging to assess the value of recycling (Ekvall et al*.* 2020).

The Screening LCA also examined two further situations, performing sensitivity analyses on the impact of transportation modes for delivering recycled PC granules from recycler tin US to the manufacturer site in GB and of different electricity mixes for the recycling activities on the results (supplementary file Fig. S4 and Fig. S5). The results indicate that shifting from sea to air shipments could affect the outcomes, worsening the results for some indicators, reducing the benefits of both closed-loop and open-loop scenarios. The second sensitivity analysis showed no noticeable changes, not worsening the baseline situation.

This could reduce the incentive to recycle products in an open-loop system, which demands logistical efforts to preserve the plastic's value, especially when carbon emission savings are prioritized. However, even open-loop recycling is generally seen as environmentally beneficial.

This study focused on evaluating the technical feasibility of closed-loop recycling in life science applications, without considering logistics and business factors. To advance recycling of small-scale PC vessels post-use, these aspects must be addressed. The trials were conducted under ideal sorting conditions, with all recycling steps performed by experts. Costs were not assessed, and the value of recycled PC will depend on collection and sorting costs (Nzihou et al*.* 2022).

A major challenge is creating a specific flow for used small-scale vessels, ideally separating them from other waste at the point of use. While this study achieved ideal sorting quality, replicating this for commercial recycling may be difficult, especially to gather the mass needed, typically over 1 ton. Extended storage might be necessary, particularly in closed-loop processes that require complete traceability to minimize variability and contamination risks from uncontrolled material flows.

Open-loop recycling could act as an intermediate step, but the return rate must be significantly higher than current literature suggests to achieve notable carbon footprint reduction, currently the main driver for industry circularity. The European Commission directive on plastic packaging (Directive on packaging and packaging, 1994) requires Member States to establish systems for returning, collecting, reusing, and recycling used packaging to meet recycling targets. It also mandates Extended Producer Responsibility schemes for packaging. This directive is expected to increase demand for recycled plastic, spur new technology development, and foster new business models, making open-loop recycling more appealing and expanding opportunities for closed-loop recycling in life science and healthcare applications.

## Conclusion

Overall, this study underscores the potential for integrating circularity into biopharmaceutical processes, paving the way for more sustainable practices that align with industry goals for carbon footprint reduction and environmental stewardship. Achieving meaningful circularity will ultimately require collaboration across the plastics and life-science industries, supported by clear regulatory frameworks, investment in collection and sorting processes, and scalable business models.

Our results suggest that recycling PC vessels from small-scale single-use bioreactors for reuse in the same application is technically feasible. Under idealized conditions absent of potential challenges posed by material cross-contamination or non-recommended processing conditions, vessels made entirely from recycled PC performed comparably to virgin vessels in cell culture: no discernible impact on cell growth and only a few minimal effects across numerous product-quality attributes in a mAb and a bsAb, despite minor changes in plastic properties and color. Potential quality concerns from culture residues or chemical decontaminants were addressed via trace element and extractables analyses. Closed-loop recycling reduces incineration-related emissions and virgin material use. Emissions related to transport mode, however, are significant and can offset benefits from avoiding disposal or production of virgin material. Establishment of closed loops therefore requires efficient collection and processing steps. Although open-loop recycling offers limited benefit over current linear disposal routes, it can serve as an interim step to integrating recycled PC streams into high-value recycling streams and help accelerate implementation. Key hurdles to achieving closed loops for bioreactors include: a need for dedicated pathways for discarded bioreactor vessels, sufficient volumes for commercial recycling, and a reliable flow from collection and sorting to reintegration, especially given small lab-scale volumes.

Looking ahead, extending our work to further study increasingly realistic scenarios encompassing mixed materials or examining bioreactor performance after multiple recycling loops can further shed light on inevitable challenges faced in an actual closed-loop scenario. Furthermore, a cradle-to-cradle life-cycle assessment using primary industrial-scale data is needed to validate environmental results. Progress will require cross-industry collaboration, clear regulatory frameworks, investment in collection and sorting, and scalable business models, aligning with carbon-footprint and stewardship goals.

## Supplementary Information

Below is the link to the electronic supplementary material.

ESM 1 (PDF 637 KB)

## Data Availability

All data supporting the findings of this study are available within the paper and its Supplementary Information. For confidentiality and business risk it is impossible to be more specific in the description of the antibodies, but this does not impact the objectives and conclusion of the paper.

## Footnotes

1 Although a waste hierarchy is clearly established, particularly in Europe (European Parliament, Council of the European Union (2008), there are still some discussions on recycling definitions and hierarchy. Mechanical recycling is generally considered the most desirable approach since it is expected to have the best emission and economical profile (European Commission et al., (2019); European Commission et al., (2023).

2 Ambr® Vessels Sustainability Fact Sheet https://www.sartorius.com/download/1676714/ambr-sustainability-fact-sheet-brochure-en-b-pdf-data.pdf.

3 As defined by the US National Institute for Occupational Safety and Health.

4 For confidentiality and business risk it is impossible to be more specific in the description of the antibodies, but this does not impact the objectives and conclusion of the paper.

5 Operational replicates were suitable to investigate vessel to vessel variability and necessary under material constraints. Biological variability between batch to batch from established cell lines is negligible.
