# Supplementary material for: Lab Scale Closed-Loop Recycling of Polycarbonate Bioreactors for Sustainable Process Development
Source: Appl Microbiol Biotechnol. 2026 Mar 27;110:127. doi: 10.1007/s00253-026-13796-z (PMC13032987; doi:10.1007/s00253-026-13796-z)
Supplement: Supplementary file 1 [file 253_2026_13796_MOESM1_ESM.pdf]

Lab Scale Closed-Loop Recycling of Polycarbonate Bioreactors for Sustainable Process Development

Supplemental information

Material and methods

Extractables

- High-Performance Liquid Chromatography (HPLC) with Ultraviolet (UV) detection was performed using Agilent 1200 system, featuring a G 1314A VWD detector and a C18 Nucleosil column (5 µm, 250 mm/4.6 mm) with a Nucleosil C18 100-5 guard column.
- Gas Chromatography-Mass Spectrometry (GC-MS) was performed using a Clarus 600GC and Clarus 600T MS Turbo, with a USP G27 column and electron ionization (EI) at 70 eV.
- Ultra-Performance Liquid Chromatography–High-Resolution Mass Spectrometry (LC-MS) was conducted using a Waters ACQUITY UPLC I-Class system with a Xevo G2-XS ESI-QTOF mass analyzer and a C18 column (BEH, 1.7 µm, 2.1 mm × 100 mm) with a guard column.

Cell Culture and monoclonal antibody (mAb and bsAb) production

- Culture growth and metabolite levels were monitored via integrated sampling on a BioProfile FLEX2 (Nova Biomedical, Waltham, MA, USA) with a cell density and viability (CDV) module.
- ProA titer was measured via high-performance liquid chromatography (HPLC) with an Agilent 1260 Infinity HPLC (Agilent Technologies, Santa Clara, CA, USA).
- ProA purification was carried out using a Tecan Freedom EVO liquid handler (Tecan Group Ltd., Männedorf, Switzerland).
- UP-SEC, IEX, and N-glycan (using an InstantPC protocol) were carried out using a Waters UPLC H Class (Waters Corporation, Milford, MA, USA). The icIEF analyses used a Maurice icIEF Cartridge and capillary electrophoresis instrument (ProteinSimple, San Jose, CA, USA). CE-SDS was performed using a LabChip GXII (Revvity, Inc., Waltham, MA, USA).

Trace element analysis

- Ultrapure, sterile water was attained from a Milli-Q Q-POD with a 0.22 µm Millipak filter (Millipore Sigma, Burlington, MA, USA).
- Vessels were incubated on an Ambr® advanced microscale bioreactor workstation (Sartorius, Goettingen, Germany), kept sterile by a Baker SterilGARD e3 biological safety cabinet (The Baker Company, Inc., Sanford, ME, USA).
- Reactor contents were poured into 15 mL Falcon tubes (Corning, Inc., Corning, NY, USA) and stored at 4 degrees Celsius for two weeks before ICP-MS testing.
- Trace element concentrations were detected and calculated against a 5-point calibration curve on an Agilent 8900 Triple Quadrupole ICP-MS (Agilent Technologies, Inc., Santa Clara, CA, USA).
- Linear fit and one-way ANOVA analyses were carried out using JMP statistical software (SAS Institute, Cary, NC, USA).

Life Cycle Assessment (LCA)

- The goal of this Screening LCA is to investigate potential environmental improvement solutions for the product under study, focusing on the recycling perspective.
- A declared unit was chosen instead of a functional unit because the function of the Ambr® 250 HT vessels is subject to many variables and to different cases according to the customers that are using it. In this case, the reference flow corresponds to the declared unit, that is one 250 mL bioreactor vessel with packaging.
- The use phase was excluded from the calculations due to high variability according to wide customers' applications.
- As regards allocation, it is avoided by dividing the process into sub-processes and collecting the inventory data for each sub-process. As regards overhead electricity for operations, an allocation according to the total mass produced by the plant has been adopted.
- As regards End of Life allocation, the cut-off approach was adopted. In the case of the closed-loop scenarios, where the exhausted vessel is recovered and recycled to become secondary raw material to be used for the same product, the processes related to the collection and the recycling activities were considered. In the open-loop scenario, where the polycarbonate is not recycled in the same product, the processes of recycling were considered out of the system boundaries of the study.
- As regards the mass balance for the calculation of the theoretical value of 90% of recycled content of the vessel, the weight of the vessel was considered (around 76 g) as well as the percentages of scrap/waste during recycling processes (1.5%) and vessel production (5% during injection moulding and 1% during quality testing). According to the hypotheses made, the

vessel available at the end of life is providing sufficient polycarbonate for the raw material needed to produce a new vessel with a maximum of 90% of recycled content. Different conditions could lead to different outcomes.

- The Life Cycle Inventory for the main processes is shown in Table S1.

**Application**		**Datasets**	**Database**	**Process**
Materials	Production of product	(DE) Polycarbonate granulate (PC) and injection moulding	Sphera	Lid, drive shaft, vessel, pipette cap
		(DE) Polypropylene granulate (PP) mix and injection moulding	Sphera	Impeller, pin, manifold, tube support
		(DE) Ethylene Propylene Diene Elastomer (EPDM) and injection moulding	Sphera	O-rings
		(DE) Styrene ethene butene styrene copolymer (SEBS, TPS, TPE-S) and others	Sphera/ ecoinvent	TPE tubing
		(RER) Polyethylene low density granulate (LDPE/PE-LD) and injection moulding	Sphera	Sparger, filter, pipette cap seal
		(RER) Polyethylene terephthalate bottle grade granulate (PET) via PTA and others	Sphera	Sensor
		(DE) Polyethylene Film (PE-LD) without additives	Sphera	Labels
		(RER) Kraftliner 2021; by-products: tall oil, turpentine; cut-off EoL; [mass allocation] and (RER) Corrugated board 2021; excl. paper production; input: paper	Sphera	Sensor spot
	Production of packaging	(RER) market for EUR-flat pallet	ecoinvent	Pallet
		(RER) core board production	ecoinvent	Divider/spacer
		(RER) corrugated board box production	ecoinvent	Box
		(GLO) market for printed paper	ecoinvent	Leaflet
		(RER) Polyethylene film (PE-LD)	Sphera	Film
Transport		(US) Diesel mix at filling station	Sphera	Fuels and means of transport for raw material upstream transportation, product distribution and takeback
		(RER) Diesel mix at filling station	Sphera	
		(GB) Diesel mix at filling station	Sphera	
		(DE) Diesel mix at filling station	Sphera	
		(GLO) Truck, Diesel, Euro mix, 7.5 - 12t gross weight	Sphera	
		(GLO) Truck, Diesel, Euro V, 28 - 32t gross weight	Sphera	
		(GLO) Truck, Diesel, Euro V, more than 32t gross weight	Sphera	
		(RER) Heavy fuel oil at refinery (1.0wt.% S)	Sphera	
		(GLO) Container ship, 5,000 to 200,000 dwt payload capacity, deep sea	Sphera	
		(RER) Kerosene / Jet A1 at refinery	Sphera	
		(GLO) Cargo plane, 65 t payload	Sphera	
		(GLO) Cargo plane, 113 t payload	Sphera	
Energy		(US) Electricity grid mix – RFCW	Sphera	Recycling
		(GB) Residual grid mix	Sphera	At manufacturer
		(DE) Residual grid mix	Sphera	E-beam sterilization
		(US) Electricity grid mix – RFCE	Sphera	Sterilization of the product at the End of Life
Waste		(RER) Polycarbonate (PC) in waste incineration plant (0% H2O content)	Sphera	End of Life of components in polycarbonate
		(RER) Plastic packaging in municipal waste incineration plant (0% H2O content)	Sphera	End of Life of all the other product components and of plastic packaging
		(RER) Plastic waste on landfill	Sphera	End of Life of plastic packaging
		(RoW) treatment of waste paperboard, municipal incineration	ecoinvent	End of life of paper/cardboard packaging
		(RoW) treatment of waste paperboard, sanitary landfill	ecoinvent	
		(RoW) treatment of waste wood, untreated, municipal incineration	ecoinvent	End of life of wood packaging
		(RoW) treatment of waste wood, untreated, sanitary landfill	ecoinvent	

Table S1 - The Life Cycle Inventory for the main processes

- As regards data quality, the baseline and the two scenarios assessed share the same datasets and hypotheses for all the stages except for the end of life. For the end of life of the two scenarios addressed, the vessel is involved in a closed-loop recycling.
- Being aware of the high uncertainty at the level of the End of Life, the options chosen in the scenario analysis are close to the opposites, including a low percentage of recycled content (20%) and the potential theoretical maximum percentage of recycled content (90%) that could be achieved without requiring additional polycarbonate waste coming from other products.
- As regards mass balance for the calculation of the theoretical value of 90% of recycled content of the vessel, the weight of the vessel was considered (around 76 g) as well as the percentages of scrap/waste during recycling processes (1.5%) and vessel production (5% during injection moulding and 1% during quality testing).

Results

Cell Culture and monoclonal antibody (mAb and bsAb) production

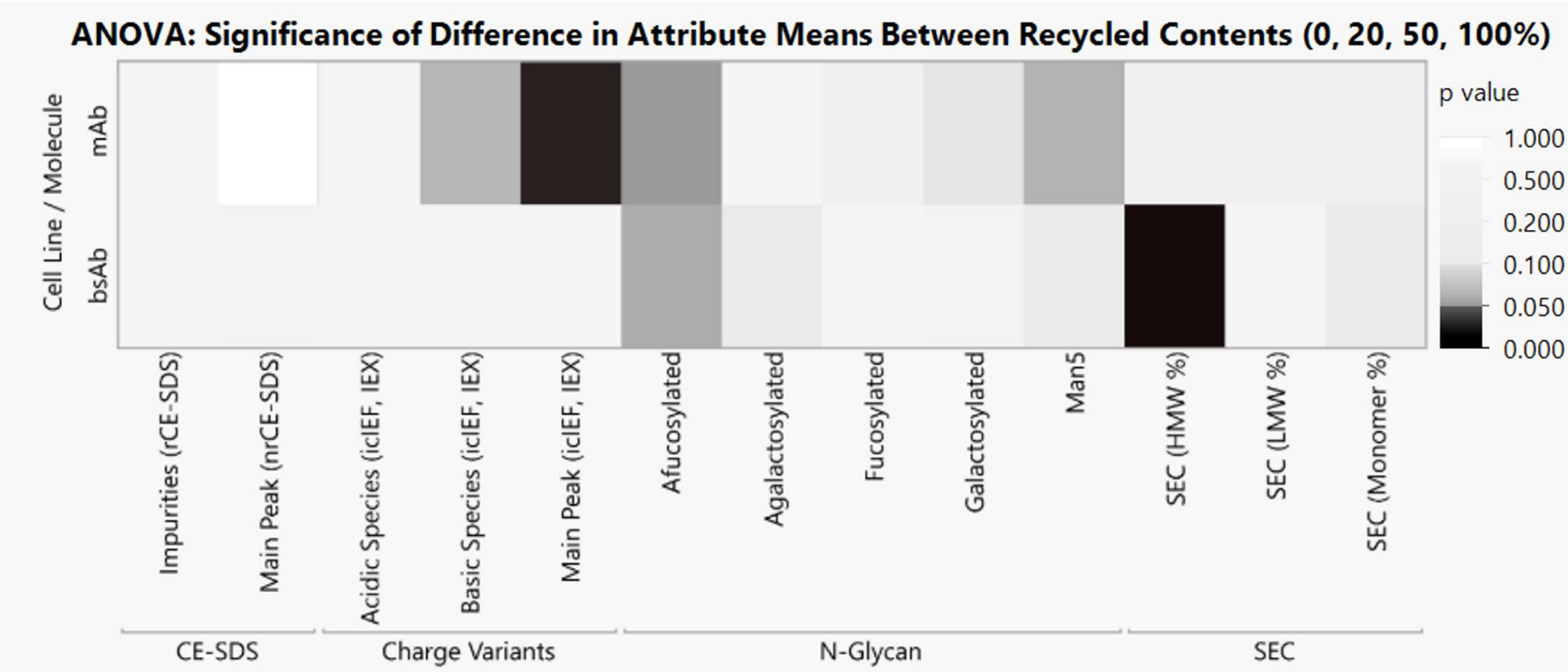

Fig. S1 One-Way ANOVA with % recycled treated as a categorical variable comparing attribute means between 0, 20, 50, and 100% recycled vessels. P values are visualized according to the grayscale gradient. Black regions indicate significance of p-values ($p < \alpha = 0.05$) of the difference in attribute means between vessel recycling contents.

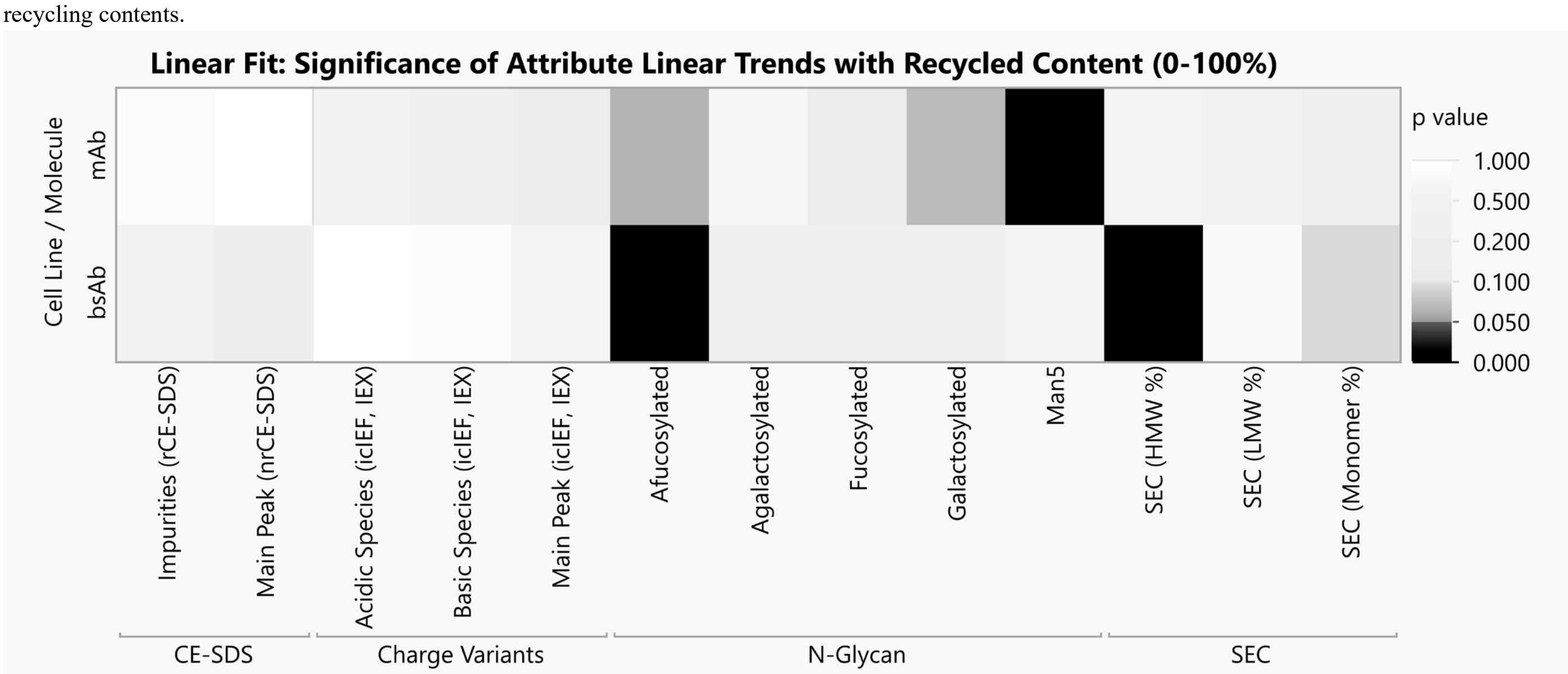

Fig. S2. Linear fit model with % recycled treated as a continuous variable from 0 to 100%. P values are visualized according to the grayscale gradient. Black regions indicate significant p-values ($p < \alpha = 0.05$), suggesting a non-zero sloped linear trend between quality attribute and recycled content.

Discussion

Environmental impact

Using recycled content may not always be feasible. Therefore, two additional scenarios were included in the analysis (Fig. S3)

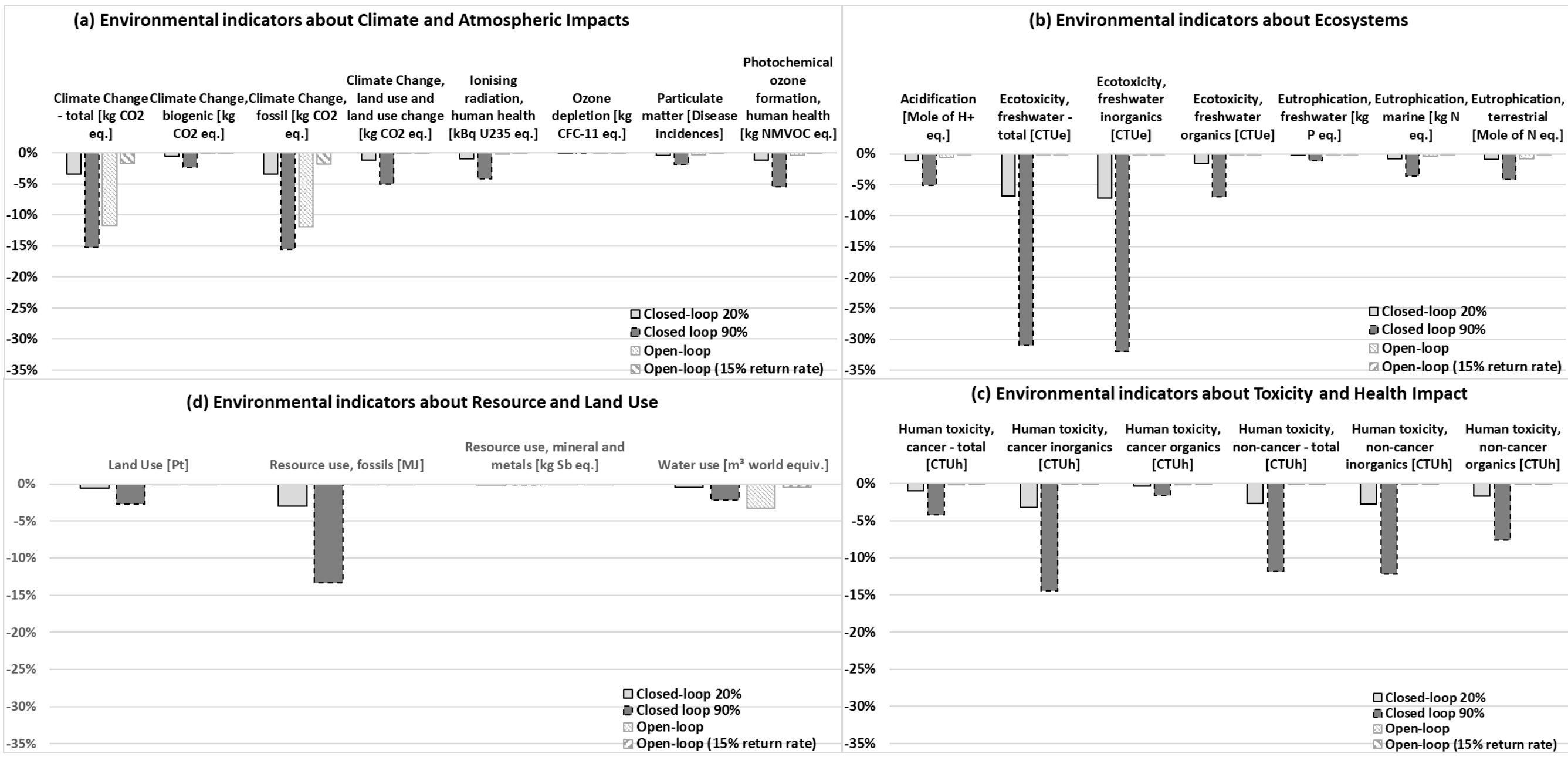

Fig. S3 Difference between the baseline and the scenarios analyzed: Closed-loop – 20% and Closed-loop – 90%, Open-loop, Open-loop (15% return rate). Life Cycle Impact Assessment results obtained by applying the EF v3.1, grouped as follows: (a) Climate and Atmospheric impacts, (b) Ecosystems, (c) Toxicity and Health Impacts, (d) Resource and Land Use. Mode of transport: ship and truck.

A hotspot analysis was performed highlighting that, for most of the impact categories, the incidence of the PC vessel in terms of raw materials, processing and End of Life, does not exceed 20%.

Being aware of the high uncertainty related to the variables at play at the End-of-Life level, two sensitivity analyses were performed acting on:

- changing from sea shipments to air shipment for the transportation of the recycled granules from the recycler in the US to the manufacturer site in the UK (Fig. S4)
- changing the electricity mix for the recycling activities, comparing US electricity mix (RFCW) used in the baseline with German and European residual mixes (Fig. S5)

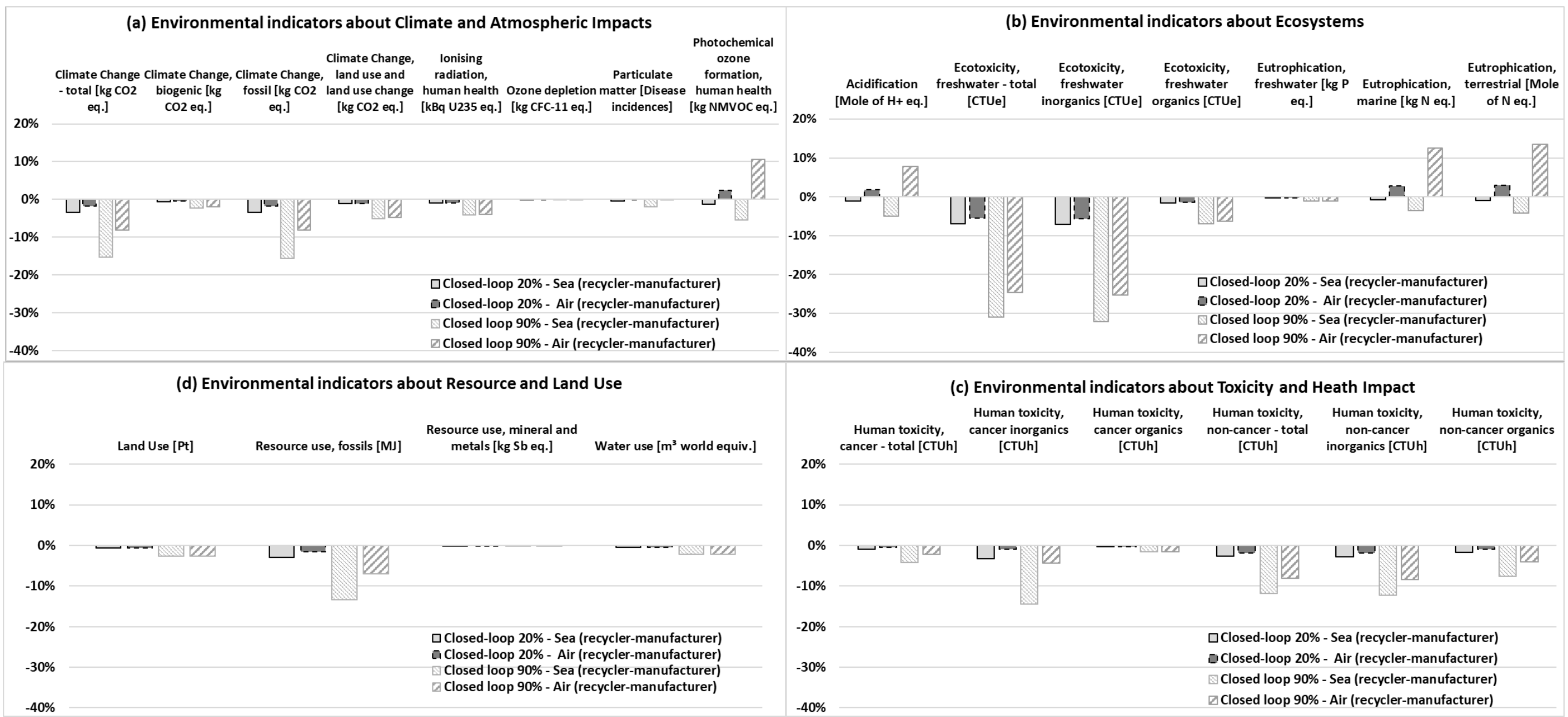

Fig. S4 Sensitivity analysis. Difference between the baseline and: analyzed scenarios Closed-loop – 20% via sea from the recycler to the manufacturer, Closed-loop – 20% via air from the recycler to the manufacturer; Closed-loop – 90% via sea from the recycler to the manufacturer; Closed-loop – 90% via air from the recycler to the manufacturer. Life Cycle Impact Assessment results obtained by applying the EF v3.1, grouped as follows: Climate and Atmospheric impacts, Ecosystems, Toxicity and Health impacts, Resource and Land Use.

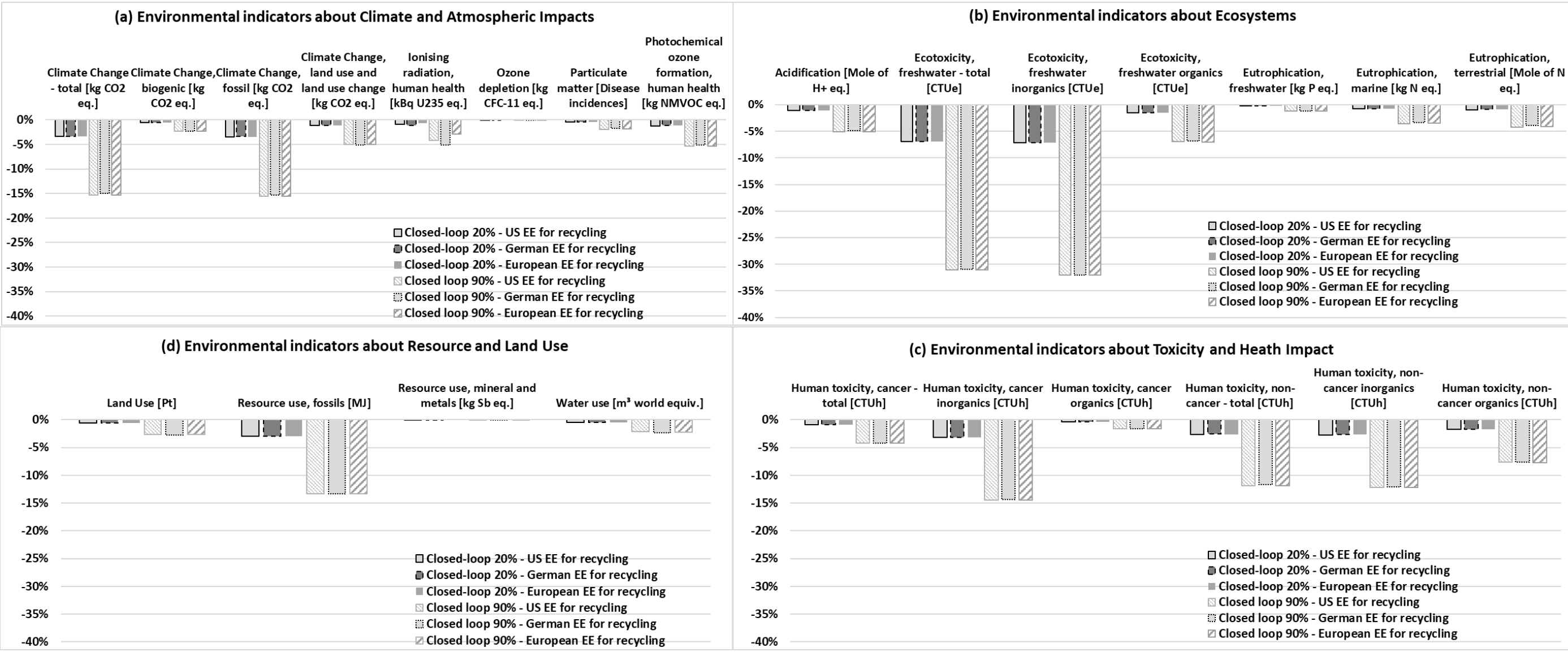

Fig. S5 Sensitivity analysis. Difference between the baseline and: analyzed scenarios Closed-loop – 20% with US electricity for recycling, Closed-loop – 20% with German residual electricity mix for recycling; Closed-loop – 20% with European residual electricity mix for recycling; Closed-loop – 90% with US electricity for recycling; Closed-loop – 90% with German residual electricity mix for recycling; Closed-loop – 90% with European residual electricity mix for recycling. Life Cycle Impact Assessment results obtained by applying the EF v3.1, grouped as follows: Climate and Atmospheric impacts, Ecosystems, Toxicity and Health impacts, Resource and Land Use.
